# Advances of Vibrational Spectroscopic Technologies in Life Sciences

**DOI:** 10.3390/molecules22020278

**Published:** 2017-02-13

**Authors:** Christian W. Huck

**Affiliations:** Institute of Analytical Chemistry and Radiochemistry, CCB-Center for Chemistry and Biomedicine, Innrain 80/82, 6020 Innsbruck, Austria; christian.w.huck@uibk.ac.at; Tel.: +43-512-507-57304

Generally, vibrational spectroscopy enjoys increasing popularity [1]. One reason for this is its potential for fast, non-invasive and simultaneous analysis of different chemical and physical parameters and its applicability for both quantitative and qualitative analysis [2]. Another reason is the fast advancement of technical and theoretical methodologies [3]. Life sciences, including food, proteomics, human medicine, pharmacy, therapy, bacteriology, organic synthesis and herbal medicines are only some disciplines to be mentioned among others taking direct advantages of employing vibrational spectroscopy [4,5,6,7,8,9]. During the last fifteen years the number of publications containing the concept of vibrational spectroscopy has tripled. Vibrational spectroscopy comprises mid infrared (MIR), near infrared (NIR) and Raman spectroscopy, also called the “three sisters”. MIR is based on the change of the irritated molecules dipole moment resulting in fundamental vibrations [10]. In NIR spectroscopy excitation to higher electronic states are enabled according to the model of the an-harmonic oscillator resulting in overtone and combination vibrations. Raman spectroscopy uses the effect of inelastic scattering of monochromatic light, which is also termed as “Raman effect” [11]. 

Miniaturization of spectrometers in size down to portable and handheld devices for all three vibrational modes makes them more popular even outside the spectroscopic community [12,13,14]. Especially, NIR spectrometers have made an immense technical advancement due to the introduction of micro electro mechanical systems (MEMS) and linear variable filters (LVF) [14]. Fast imaging/mapping devices enable an efficient quality control with spatial resolution [15]. But it is not only the technical but also the theoretical progress making all three techniques more suitable for different life science related questions. On one side chemometrical and multivariate approaches are permanently advancing. On the other side quantum chemical calculations, especially the employment of modern an-harmonic vibrational analysis for the modeling of non-fundamental vibrational transitions in NIR spectra of relative complex molecules, are extremely helpful for understanding the structure-spectra correlations in NIR region [14]. Within this special issue it was the intention to draw a selective bow between technical development and high-tech applications. Therefore, I hope that it is of high interest for a broad readership in the field of life science research.

This special issue contains fifteen published papers, twelve research and three review articles. Let me first outline the principal content of the published research articles in chronological sequence: Eisenstecken, Oberhuber et al. described the potential of NIR spectroscopy for predicting quality parameters of Cripps Pink and Braeburn apples such as total soluble solids (TSS), acidity (TA), firmness, and individual sugars (glucose, fructose, sucrose and xylose) [16].

Baldassarre, Barth et al. described an approach for the simultaneous fitting of MIR absorption spectra for an improved analysis of proteins. The approach termed as “co-fitting” leads to a more reliable modeling of the experimental data because it uses more spectral information than the standard approach of fitting only the absorption spectrum and it also avoids that the fitting routine becomes trapped in local minima. The approach was tested with different mixtures of α/β proteins containing ribonuclease A, pyruvate kinase and aconitase [17].

Schuetz, Masic et al. investigated the anisotropy in bone demineralization by high intensity synchrotron-based far-IR spectroscopy (50–500 cm^−1^) to gain new insights into structure and chemical composition of bovine fibro lamellar bone [18]. 

Murayama, Ozaki et al. critically evaluated the efficiency of a portable NIR imaging device based on a polychromator type spectrometer operating at high-speed and high-resolution photo diode array detector for monitoring a pharmaceutical blending process [19].

Achata, Gowen et al. published a study on the application of NIR hyperspectral chemical imaging for monitoring moisture content and water activity in low moisture systems as they are key parameters in predicting the stability of low moisture content products. NIR hyperspectral chemical imaging was shown as an emerging technique for spatially characterizing the spectral properties of samples [20].

Prieto-Calvo, Alvarez-Ordonez et al. evaluated the feasibility of FTIR spectroscopy and microscopic imaging techniques to monitor the effect of high hydrostatic pressure (HPP) as a novel food processing technology on the composition of *Escherichia coli* [21].

Lutz, Huck et al. prepared an article about the vibrational self-consistent field (VSCF) treatment calculations and the effect of grid densities [22].

Aydin published a comparative study about the structural and vibro-electronic properties of porphyrin and its derivatives employing density functional theory (DFT) and Raman spectroscopy [23].

Lewis, Blake et al. investigated the dynamic light scattering and Raman spectroscopy approach for characterizing the aggregation of therapeutic proteins. The ability to differentiate between unfolding and aggregation that the combination of both dynamic light scattering (DLS) and Raman spectroscopy provides enables for the first time insights into underlying protein aggregation mechanisms [24].

Campos, Stephani et al. investigated the quality of frankfurters by FT-Raman spectroscopy. A multivariate classification model was developed to identify the Frankfurter type, including chicken, turkey and mixed meat. The established PLS-DA models give sensitivity and specificity values on the test-set in the ranges of 88%–100%, showing high performance [25].

Farias and Carneiro described the application of a novel Raman spectroscopic method for the simultaneous quantification of three polymorphic forms of carbamazepine in the presence of excipients. This method was shown being highly useful because it can affect the bioavailability of the final product [26].

Sandasi, Viljoen et al. introduced a novel hyperspectral imaging and chemometric modeling approach for the quality control of the herbal medicine *Echinacea*. Authors explained how this technique can be used to differentiate between the root and leaf samples of three different species and how this method can be applied for differentiation between different root species [27].

Furthermore, the special issue also contains three review articles providing an excellent overview about selected topics. The first review authored by Huck summarizes the recent development in solid-phase extraction (SPE) methods that can be used in combination with NIR and attenuated total reflection (ATR) spectroscopy [28]. Cozzolino prepared an excellent overview about the role of visible and infrared spectroscopy combined with chemometrics to measure phenolic compounds in grape and wine samples. The content of phenolic compounds determines the state of phenolic ripening of red grapes, which is a key criterion in setting the harvest date to produce quality red wines. NIR and MIR spectroscopy are described offering high potential to simplify and reduce the analytical time for a range of grape and wine analytes [29]. Finally, Jabeen, Bonn et al. have reviewed Au-nanomaterials as a superior choice for NIR photothermal therapy [30].

In summary, in this special issue on one side novel technical and theoretical attempts have been introduced including miniaturization of spectrometers and quantum mechanics, on the other side highly selective applications have been introduced in various fields of life science related research. From those articles and especially the three review articles the reader could build up not only his own mind upon the current state of the art, but also might get stimulated to get his own idea for new developments and approaches.
